# Evaluation of Crimean-Congo Hemorrhagic Fever Orthonairovirus AviTagged Nucleoprotein for Potential Application in Diagnosis

**DOI:** 10.29252/ibj.23.6.379

**Published:** 2019-11

**Authors:** Tahmineh Jalali, Mostafa Salehi-Vaziri, Mohammad Hassan Pouriayevali, Seyed Latif Mousavi Gargari

**Affiliations:** 1 Department of Biology, Faculty of Basic Sciences, Shahed University, Tehran, Iran;; 2 Department of Arboviruses and Viral Hemorrhagic Fevers (National Reference Laboratory), Pasteur Institute of Iran, Tehran, Iran;; 3 Reaserch Center for Emerging and Reemerging Infectious Diseases, Pasteur Institute of Iran, Tehran, Iran

**Keywords:** Crimean congo hemorrhagic fever, Enzyme-linked immunosorbent assay, Nucleoprotein

## Abstract

**Background:**

Crimean-Congo hemorrhagic fever (CCHF) is an acute viral zoonotic disease, with a mortality rate of 30-50%. There is no approved vaccine or any specific antiviral treatment for CCHF; therefore, the rapid diagnosis seems to be crucial for both efficient supportive therapy and control of infection spread. In this study, the potency of recombinant nucleoprotein of virus expressed in prokaryotic system was investigated for diagnosis of the infection.

**Methods:**

The DNA sequence of complete nucleoprotein ORF was codon optimized based on *E. coli* codon usage and synthesized commercially. The gene was subcloned in pCA4 vector and expressed in *E. coli* BL21 (DE3). Refolding and simultaneous purification of nucleoprotein were performed using protein folding liquid chromatography method. The recombinant nucleoprotein was analyzed by Western blotting, ELISA, immunofluorescence assay, and circular dichroism. Forty eight human samples, in three IgM positive and three negative control groups, were evaluated using recombinant nucleoprotein in a capture ELISA setting. Serum from healthy individuals, those suspected to viral hemorrhagic fevers, and positive samples of Chikungunya and Dengue were considered as negative controls.

**Results:**

The existence and structure of recombinant nucleoprotein were verified and confirmed. Capture IgM ELISA detected all positive samples (sensitivity of 100%), but none of the 25 negative samples was detected as positive (specificity of 100%). The test also detected all the included genotypes of virus.

**Conclusion:**

Our recombinant nucleoprotein can be used in IgM capture ELISA for easy and efficient detection of CCHF in any lab in endemic regions.

## INTRODUCTION

Crimean-Congo hemorrhagic fever (CCHF) is an acute, viral, zoonotic disease, with a mortality rate of 30-50%, considered as one of the infectious pathogens with a high risk of epidemic in near future^[^^1^^,^^2^^]^. The causative agent of the disease is one of the most important viruses classified under *Nairoviridae* family and *Orthonairovirus* genus carried and spread by tick^[^^3^^,^^4^^]^. Despite its high death rate and transmissibility among human beings, currently there are no approved vaccines or specific therapeutics against this virus. Therefore, rapid diagnosis of the disease seems to be crucial for both effective treatment of patients and control of infection transmission. It worth to mention that CCHF symptoms are not specific; therefore, the only way of precise diagnosis is laboratory tests. 

The routine diagnostic tests include virus’s genome detection by RT-PCR or evaluation of serum IgM by ELISA^[^^5^^]^. Although highly sensitive and specific, the genome-based detection method is usually difficult since the collection of samples in a short viremia period (one week) is problematic. This is mainly due to the occurrence of most infections in rural, far away regions. IgM, which is detectable in blood for 4-6 months, can play an important role in the diagnosis process^[6]^. Several ELISA methods have been developed to detect anti-CCHF virus IgM; many of these methods utilize inactivated whole virus particle as an antigen. BSL-4 laboratory is necessary for virus culture, which is not available in most endemic regions. The recombinant expression of necessary antigens is a possible solution for this shortcoming^[^^7^^]^. CCHF virus nucleoprotein is the most important structural protein against which the highest rates of antibodies are raised. Therefore, it can be considered as a reliable candidate for designing serological assays such as ELISA^[^^8^^]^. 

Although there are some available commercial ELISA kits to detect the specific IgM against CCHF in human sera, the highest sensitivity reported is still 87%, indicating that they can be improved^[^^9^^]^. The aim of this study was to produce a recombinant viral nucleoprotein antigen in a prokaryotic expression system, to develop a safe, low-cost and more sensitive diagnostic platform.

## MATERIALS AND METHODS

**Plasmid and reagents**

pAC4 vector (Cat No: PAC4) and AbCA-AbC mab antibody conjugated to sepharose resin (Cat No: AbCA) were purchased from Avidity, LLC. (Aurora, Colorado, USA). VectoCrimean-CHF-Ag (Cat No: D-5056) and VectoCrimean-CHF-IgM (Cat No: D-5054) were obtained from Vector-Best (Vector-Novosibirsk, Russia). *Nco*I (Cat No: ER0571) and *Bam*HI (Cat No: ER0051) were procured from Thermo Fisher Scientific Inc., USA. PVDF membrane (Cat No: 03010040001) was from Roche Diagnostics (GmbH, Germany). Extra Thick Blot Paper (Cat No: 1703966) were purchased from Bio-Rad (Bio-Rad Laboratories Inc., USA). Immune ascitic fluid against CCHF (M_A_F_4_, Institute Pasteur, Dakar, Senegal), goat anti-mouse IgG, rabbit anti-human IgM (Anti-µ-Chain; ICN, Ohio, USA [Cat Nos: 55558 and 55017]), and other chemicals were acquired from Merck and/or Sigma, USA. 

**Synthetic gene and subcloning**

**Fig. 1:**
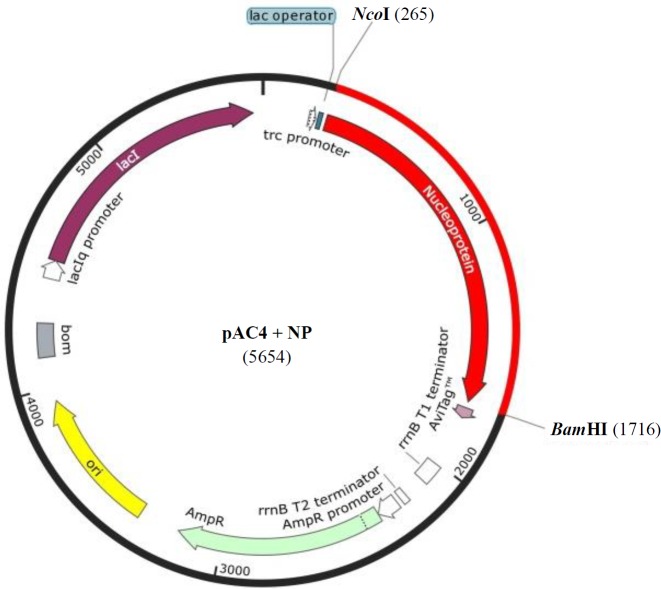
Schematic view of the cloning strategy showing the restriction sites, *Nco*I, and *Bam*HI used for cloning

 The DNA sequence of complete open reading frame of nucleocapsid protein (1454 bp) from Matin strain (accession number: AF527810.1) was codon optimized for *Escherichia coli *(BL21 [DE3]) and synthesized in pBSK(+) simple Amp by Biomatik (Cambridge, Ontario, Canada). *Nco*I and *Bam*HI restriction enzymes were included for 5’ and 3’ ends, respectively. The embedded ATG in *Nco*I restriction site (C^↓^CATGG) simplified the subsequent step of subcloning and protein expression in the right frame. The gene was subcloned in pAC4 expression vector, which adds AviTag to the C-terminus of nucleoprotein as a fusion (Fig. 1).

**Expression, purification, and refolding of recombinant nucleoprotein**

For optimal growth and recombinant nucleoprotein production, 5 ml of fresh cultured bacteria was inoculated into 500 ml of Luria-Bertani containing 100 µg/ml of ampicillin and was incubated at 37 °C in a shaking incubator at 250 rpm until OD_600_ of medium reached 0.4. IPTG with the final concentration of 1 mM was added to the culture and incubated at 37 °C for 4 hours. Medium was centrifuged at 4 °C, 5,000 g for 5 min, and the pellet was washed twice with PBS and lysed with a sonication using a lysis buffer (140 mM of NaCl, 2.7 mM of KCl, 10 mM of Na_2_HPO_4_, and 1.8 mM of KH_2_PO_4_, pH 7.3). The cell lysate was analyzed on SDS-PAGE. Inclusion bodies (IBs) were rinsed twice with the lysis buffer, followed by 2× washing with 50 mM of Tris-HCl, 2.5 mM of EDTA, 0.1% Tween-20, and then 2× washing with 50 mM of Tris-HCl, 2.5 mM of EDTA, and 1 M of NaCl. The pallet was subsequently washed 2× with 50 mM of Tris-HCl, 2.5 mM of EDTA, and 2 M of urea, and the final washing was carried out with 1× 50 mM of Tris-HCl and 2.5 mM of EDTA, followed by washing with double distilled water. To resuspend the pellet after each wash, a brief sonication was applied. The washed pellet was finally solubilized in a solubilization buffer (50 mM of Tris-HCl, 5 mM of EDTA, and 6 M of urea) by incubating at 4 °C overnight^[10-12]^. Refolding and simultaneous purification of nucleoprotein were performed with 4 ml of AbCA-AbC resin using protein folding liquid chromatography method. The column was equilibrated with 40 ml (10 CV) of PBS containing additional 50 mM of NaCl, at 4 °C. The solubilized protein was loaded on column and incubated with gentle flip flop shaking at room temperature for 2 hours. The column was washed with gradient buffers at 4 °C as follows: 15 ml of buffer 1 (50 mM of Tris-HCl, 5 mM of EDTA, 50 mM of NaCl, and 4 M of urea), 15 ml of buffer 2 (50 mM of Tris-HCl, 5 mM of EDTA, 50 mM of NaCl, and 2 M of urea), and 10 ml of PBS. The resin was incubated with 5 ml of glycine (100 mM) as the elution buffer at 4 °C for one hour, and the protein was eluted with another 5 ml of glycine. For desalting and concentrating the recombinant protein, Amicon centrifugal filter device, with a cut-off size of 30 MWCO, was used. According to device user's guide, the eluted protein was added to the device and then centrifuged at 4 °C at 4000 g for 10 min.

SDS-PAGE analysis and Western blotting of the recombinant protein

Recombinant protein was analyzed on 12% SDS-PAGE with Coomassie blue staining. For Western blotting, gel was soaked in the protein transfer buffer (25 mM of Tris-base, 198 mM of glycine, and 200 ml/L of methanol) for 15 min. PVDF membrane was activated with methanol and equilibrated in the protein transfer buffer for 15 min. The transfer sandwich was assembled according to the manufacturer’s instructions (Mini Trans-Blot SD semi-dry transfer cell, Bio-Rad, CA, USA). The band-transfer was carried out at 12 V for 25 min. Following transfer, non-specific binding sites on the membrane were blocked using the blocking buffer (5% skim milk, 25 mM of Tris, 0.15 M of NaCl, 0.1% Tween-20, pH 7.4) at room temperature for 1 hour. The blot was incubated with mouse hyperimmune ascitic fluid against CCHF virus (1: 3000 dilution, M_A_F_4_, Institute Pasteur, Dakar) at 4 °C overnight. The blot was subsequently washed five times with blotting buffer (25 mM of Tris, 0.15 M of NaCl, 0.1% Tween-20, pH 7.4). The blot was incubated with horse radish proxidase-conjugated goat anti-mouse IgG (ICN ,Cat No: 55558), for 1 hour, then was washed with the blotting buffer. Next, the enzyme substrate 3-3’diaminobenzidine (DAB) solution (0.007 g of DAB, 10 ml of PBS, 8 µl of H_2_O_2_) was added to the blot and incubated in darkness until the desired band intensity was appeared^[13]^.

**Circular dichroism (CD) analysis**

Secondary structure of the recombinant nucleoprotein was analyzed with CD system. Nucleoprotein (0.3 mg/mL) in 50 mM of Na_3_PO_4_, pH 7.5, was tested in the CD system. Spectra were recorded in 180-260 nm on a circular dichroism spectrometer Model 215, Aviv, USA at a scan speed of 1 nm/s. The test was performed in triplicate, and the results were analyzed by CDNN software (Circular Dichroism analysis using Neural Networks).

**ELISA detection of nucleoprotein**

In order to detect the recombinant nucleoprotein, VectoCrimean-CHF-Ag ELISA kit (Vector-Best, Russia) in which the antibodies against CCHF virus are coated on the plate, was used. Recombinant nucleoprotein was added instead of sera sample. Three different dilutions of nucleoprotein (7, 0.7, and 0.07 mg/ml in PBS) were added to the wells, and the rest of the reaction was performed according to kit’s manual. Briefly, 100 µl from each dilution of nucleoprotein was added to each well, along with positive and negative controls, and the ELISA plate was incubated at 37 °C for 60 min. After washing, 100 µl of anti-CCHF antibodies labeled with HRP was added and incubated at 37 °C for another 60 min. 3,3’,5,5’-tetramethyl-benzidine substrate was added to the wells and incubated for 15 min. The reaction was stopped by the addition of 100 μl of 4N H_2_SO_4_, and the OD_450 _value was recorded.

**Immunofluorescence assay**

The recombinant nucleoprotein was coated on wells at 37 °C for 1 hour. Unspecific binding sites were blocked using 1% BSA at 37 °C for two hours. Mouse hyperimmune ascitic fluid against CCHF virus solution in phosphate buffered saline (1:600) was added and incubated at 37 °C for 45 min. Wells were washed five times with PBST. Anti-mouse IgG conjugated with fluorescein-5-isothiocyanate solution in PBST (1:1000) was added and incubated at 37 °C for 30 min. Wells were washed five times and observed under an Axiovert 200M inverted microscope, Zeiss, Germany.

**Diagnostic value of nucleoprotein for detecting IgM antibody**

The possibility of using the recombinant nucleoprotein to detect CCHF-specific IgM in ELISA tests was studied. A panel of three IgM-positive groups and three negative control groups were selected. Based on the result of IgM ELISA obtained by VectoCrimean -CHF-IgM kit, three positive groups were sorted as weak, medium, and high immune response. Negative control groups included healthy group, suspected to viral hemorrhagic fever, and the group of Chikungunya- and Dengue-positive samples (other frequent arboviruses in Iran and neighboring countries). All the samples in the panel were tested in parallel with molecular and serological methods to confirm positive and negative results. The CCHF virus strain in all the positive samples were sequenced and determined. The CCHF virus contains seven district clades with a relatively high diversity at the nucleotide and amino acid levels. Iran is a miniature model of the world for genotypes of CCHF virus^[^^14^^]^. The serum samples were chosen in a way to cover all the circulating CCHF genotypes in Iran: AsiaI, AsiaII, Kerman-22, EuropeI, and EuropeII^[^^1^^,^^12^^,^^15^^]^. In IgM capture ELISA test, the wells were coated overnight with diluted anti-µ chain in PBS (1:900). For each sample, two wells were assigned, test well and control well. Wells were washed with PBST (Tween-20, 0.3%) three times and blocked with PBSTM (PBST containing 3% skim milk) for 1 hour. Diluted serum in PBSTM (1:100) was incubated at 37 °C for 45 min and after washing with PBST recombinant nucleoprotein (40 µg/mL) or clear cell lysate from uninduced culture, it was incubated at 37 °C for 100 min in test or control wells, respectively. Mouse hyperimmune ascitic fluid against CCHF virus solution in PBSTM (1:600) was added to the wells and incubated at 37 °C for 45 min. Mouse anti-IgG HRP conjugate in PBSTM (1:350) was added and incubated at 37 °C for 45 min. After 10 minutes of incubation with 3,3’,5,5’-tetramethyl-benzidine substrate the reaction was terminated with 4 N H_2_SO_4_. Results were read in 450 nm with a reference of 620 nm. The wells were then washed three times with 300 µl of PBST and dried after each step. The cut-off value of the test was calculated based on Lardeux *et al.*^[^^16^^]^:

Cut-off = a. x̅ + f. SD

Where *x̅* is the mean, and SD is the standard deviation of negative controls, a and f are two multipliers, set as a = 2 and f = 0.

Sensitivity and specificity of IgM capture ELISA were calculated as follows:

## RESULTS

**Synthetic gene and sub-cloning**

**Fig. 2:**
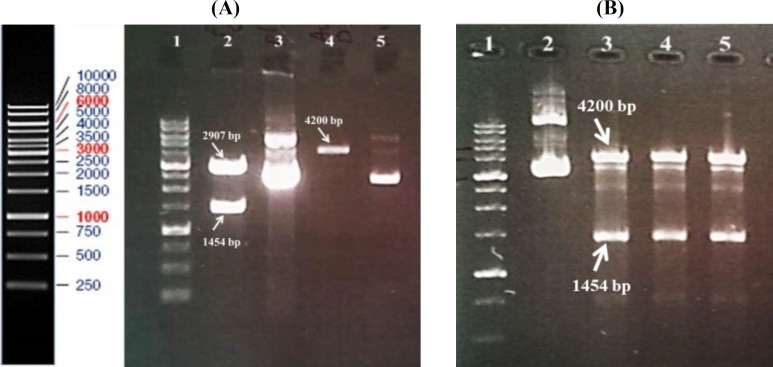
Enzyme digestion of plasmids. (A) Lane 1, 1 kb DNA ladder; lane 2, construct [pBSK (+) simple-Amp-nucleoprotein gene] digested with restriction enzymes of *Nco*I and *Bam*HI; lane 3, undigested pBSK (+) simple-Amp; lane 4, linearized pCA4 vector; lane 5, undigested pCA4. (B) Lane 1, 1 kb DNA ladder; lane 2, plasmids extracted from clones after ligation reaction; Lanes 3-5, ligate digested with *Nco*I and *Bam*HI

The codon-optimized synthetic gene (1454-bp) was delivered in pBSK (+) simple-Amp vector. The gene was sub-cloned in *Nco*I/*Bam*HI site of pAC4 expression vector (Fig. 2). The correct cloning procedure was confirmed by restriction mapping and DNA sequencing of the final vector (Fig. 2B and data not shown).

**Fig. 3:**
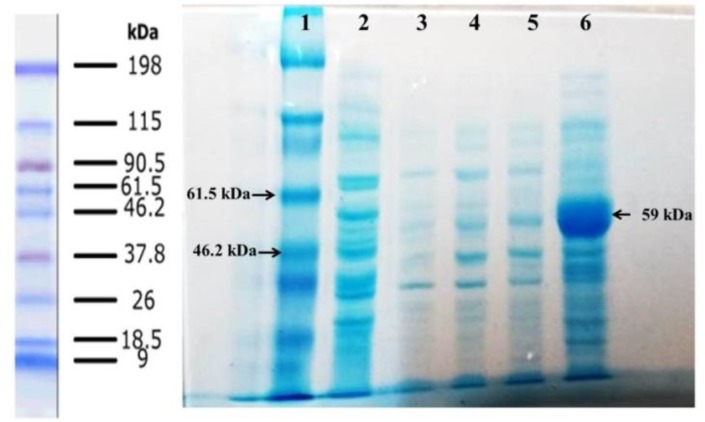
Washing steps of inclusion bodies. Lane 1, protein marker; lane 2, supernatant of second wash with lysis buffer; lane 3, supernatant of second wash with Tris EDTA Tween-20; lane 4, supernatant of second wash with Tris EDTA NaCl; lane 5, supernatant of second wash with Tris EDTA urea; lane 6, solubilized protein in solubilization buffer

**Expression, purification, and refolding of recombinant nucleoprotein**

**Fig. 4:**
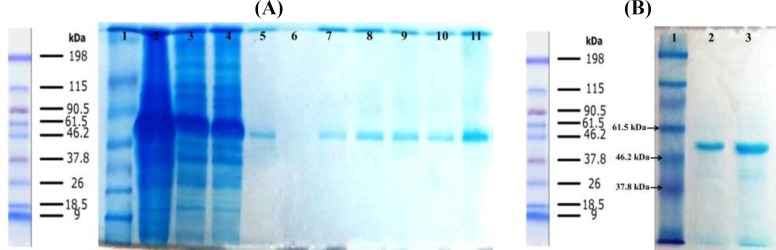
Protein purification on AbCA-AbC column. (A) lane1, protein marker; lane 2, extracted protein from cultured media before washing steps; lanes 3 and 4, inclusion bodies after washing steps; lanes 5 and 6, washing the column with refolding buffers; lanes 7-11, protein eluted from column with the elution buffer. (B) Lane 1, protein marker; lane 2 purified protein; lane 3 concentrated protein using Amicon column

The nucleoprotein was expressed under the control of the trc promoter and induced by IPTG. SDS-PAGE analysis indicated that the recombinant nucleoprotein was appeared as IB. The band on gel was matched with the predicted 59 kDa molecular weight. Figure 3 shows the steps of washing IBs, and Figure 4A indicates protein purification processes on AbCA-AbC column. The purified protein was concentrated with Amicon centrifugal filter (Fig. 4B). Refolded protein structure was examined by Western blot, Circular dichroism, ELISA, and immunofluorescence test. 

**Western blotting**

**Fig. 5:**
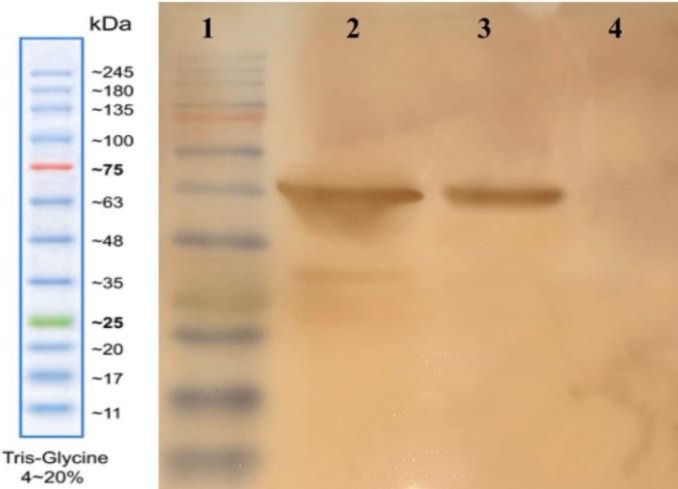
Western blot analysis. Lane 1, protein marker; lane 2, positive control (inactivated virus); lane 3, recombinant nucleoprotein; lane 4, negative control (un-induced cell lysate)

The expression of nucleoprotein was confirmed with the interaction of specific antibody against CCHF virus produced in mouse using Western blot test. Negative control, proteins extracted from *E. coli* (BL21 [DE3]) containing expression vector cultured without induction, did not show any specific band. Two samples of recombinant nucleoprotein showed visible specific bands in expected positions (Fig. 5). Positive controls were inactivated virus extracted from mouse brain in two different batches and had a clear band in accordance with the recombinant nucleoprotein. A slight difference in the size of positive controls and recombinant nucleoprotein was due to 21 amino acids added as spacer and AviTag to recombinant nucleoprotein. 

**Circular dichroism test**

**Fig. 6:**
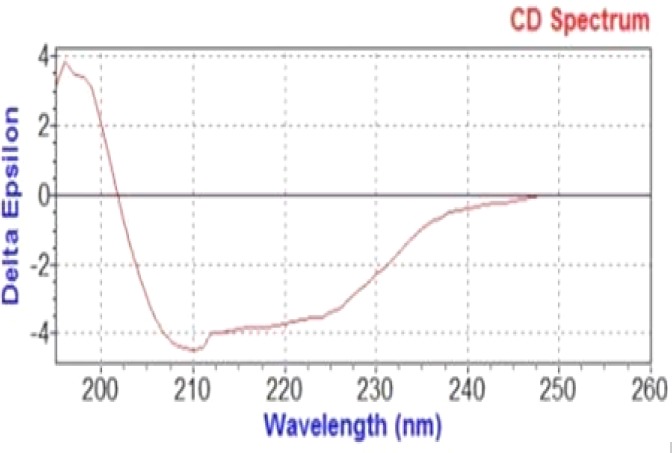
Results of CD analysis by CDNN software (Circular Dichroism analysis using Neural Networks). Nucleoprotein (0.3 mg/mL) in 50 mM of Na_3_PO_4_, pH 7.5 was tested in the CD system. Spectra were recorded in 180-260 nm on a Circular Dichroism Spectrometer Model-215, Aviv, USA at a scan speed of 1 nm/sec

CD test was performed to find the evidence of nucleoprotein secondary structure. Analyzed data with CDNN demonstrated α-helix, random coil, and β-turn percentages with the frequencies of 59.2%, 20%, and 11%, respectively (Fig. 6). 

**ELISA detection of nucleoprotein**

**Fig. 7:**
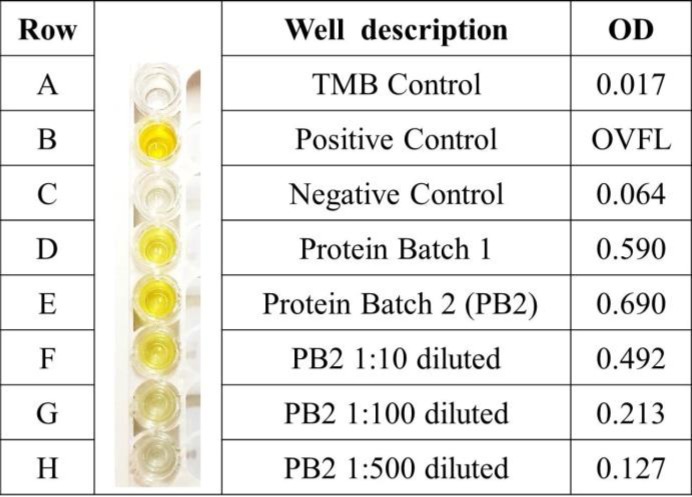
Validation of recombinant nucleoprotein by using standard antigen detection kit of CCHF

Refolded nucleoprotein in the dilutions of 1:10, 1:100, and 1:500 in PBS along with positive and negative controls were tested by using VectoCrimean-CHF-Ag. The results are exhibited in Figure 7.

**IgM capture ELISA**

Serum samples (n = 48) were tested using IgM capture ELISA. The test was blind for operator, i.e. positive and negative samples were arranged randomly. The cut-off for test was calculated to be 0.242, and capture IgM ELISA specifically identified all the positive samples. Of 23 positive samples, all were detected as positive with our ELISA test, which demonstrated 100% sensitivity, while none of the 25 negative samples were found as positive. All genotypes of the virus were detected by the test. Results are summarized in Table 1.

**Immunofluorescence assay**

**Fig. 8:**
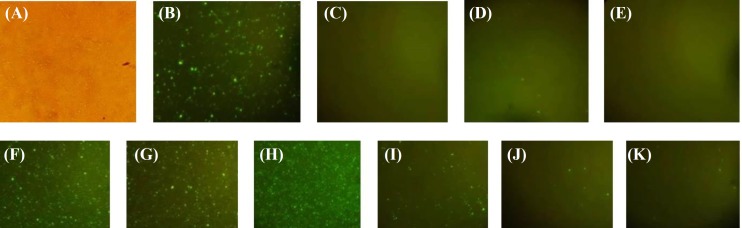
Immunofluorescence assay test results. Different concentrations of recombinant nucleoprotein is used, and description of controls are summarized as follows: (A) positive control with nucleoprotein without any dilution obtained under microscope with halogen filter; (B) experiment A repeated using the UV filter; (C) negative control coated with BSA instead of nucleoprotein; (D) negative control with ascitic fluid from non-infected mice; (E) negative control with nucleoprotein and FITC-labeled anti-mouse IgG; recombinant nucleoprotein used without any dilution (F), with 1:2 dilution in PBS (G), with 10^-1 ^dilution (H), with 10^-5 ^dilution (I), with 10^-7 ^dilution (J), with 10^-8 ^dilution (K)

Immunofluorescence assay test revealed a significant decrease in florescent intensity in accordance with coated nucleoprotein concentration. Negative controls showed no florescent dot. Results are shown in Figure 8.

## DISCUSSION

According to WHO blueprint, CCHF is among the public health threatening diseases. It bears the risk of an outbreak in near future, which can be attributed to the characteristics and epidemiology of the virus, its high genetic diversity, animal to human and human to human transmission, high mortality rates, and weak health infrastructures in endemic regions. In addition, the lack of proper measures to deal with virus or inadequacy of existing measures has introduced this disease as a major threat to humanity. With these facts in mind, one of the significant factors in counter-measures remains with the diagnostic methods^[^^17^^]^. 

In this study, a recombinant nucleoprotein was expressed in *E. coli*, and its potency for detecting specific IgM by ELISA was evaluated using positive and negative samples. Viral nucleocapsid was selected because it is the major viral protein and is detected in large amounts in the serum of infected patients. This protein also stimulates severe immune responses as an immunogen, and the majority of antibodies in serum of infected patients and livestock are raised against this protein. It is worth mentioning that viral isolates from all over the world demonstrate antigenic resemblance in their nucleocapsids, and recombinant nucleocapsid produced from S segment of any viral isolate can be used to detect all the isolates^[2,6,8]^. Problems encountered with antigen production in eukaryotic systems such as low yields and high costs make these tests infeasible in every laboratory. Therefore, the necessity of a convenient low-cost method for scale-up production is obvious. 

**Table 1:** Results of IgM capture ELISA in panel groups

**Panel group**	**Total samples**	**Genotype of ** **CCHF virus**	**Mean OD with IgM capture ELISA**		**Detected by IgM capture ELISA**	
					+ive	-ive
Positive-weak immune response	7	AsiaI-Kerman22-EuropeI-EuropeII	0.359		7	0
Positive-medium immune response	8	AsiaI-EuropeI-EuropeII	0.730		8	0
Positive-high immune response	8	AsiaI-AsiaII-Kerman22-EuropeI-EuropeII	0.980		8	0
Negative-healthy	7	NA	0.150		0	7
Negative-suspected to other VHFs	10	NA	0.113		0	10
Negative-other arboviruses (Den, Chik) IgM positives	8	NA	0.107		0	8

NA, not assigned

pAC4 vector was used for recombinant nucleoprotein expression in bacterial expression system. This vector adds the AviTag peptide (GLNDIFEAQKIEWHE) to protein C-terminal so that it can bind to biotin in this site by BirA enzyme and also interact with AbC antibody on affinity chromatography resin. In this way, nucleoprotein can be immobilized in a manageable way to carry on different studies based on biotin-streptavidin interactions. The expression vector, pAC4, contains trc promoter and the protein expression can be induced by adding IPTG. This vector is designed for high rates of expression in *E. coli*^[^^18^^]^.

Recombinant nucleoprotein expressed in *E. coli* expression system was in the form of IBs. Washing IBs in successive steps led to high purity, which had a significant positive effect on the quality and yield of final purification and refolding by affinity chromatography. Further purification and simultaneous refolding of the protein was performed using protein folding liquid chromatography strategy by affinity column of AbCA-AbC mab antibody conjugated to sepharose resin made by Avidity Company. In this column, monoclonal antibody against AviTag is fixed on agarose resins and is able to detect and bind to both biotinylated and non-biotinylated proteins. Binding AviTagged nucleoprotein to the column minimizes the aggregation of denatured proteins and enhances refolding efficiency. When denatured proteins bind to the column by their AviTag end, due to sufficient space available to each molecule to refold, hydrophobic interactions between molecules reduce, leading to the prevention of their aggregation. On the other hand, the denaturing agent, urea, is washed away gradually and passes through the column along with other proteins and impurities^[^^19^^,^^20^^]^. 

Western blot and ELISA Ag detection verified the structure of recombinant nucleoprotein after refolding. Results of nucleoprotein secondary structure analysis by CD are in good accordance with the results of Carter *et al.*^[^^21^^]^. As standard nucleoprotein was not available, conditions and buffers were set based on Carter *et al.*'s^[^^21^^]^ study, and the results were then compared. According to their reports, 66% of structure is in form of α-helix and 29% in form of random coil. Our CD results showed α-helix, random coil, and β-turn percentages of 59.2%, 20%, and 11%, respectively^[^^21^^]^. 

Recombinant viral nucleoprotein has previously been used for diagnosis of CCHF antibodies. In 1994, scientists used a bacterial expression system without codon optimization with a fusion protein to form three regions inside nucleoprotein ORF^[^^22^^]^. Emmerich *et al.*^[^^23^^]^ produced recombinant nucleoprotein conjugated with HRP, which was then used in IgM/IgG ELISA to evaluate 15 kosovar. In the present study, codon optimization was employed for more effective expression in prokaryotic system, and the whole ORF was synthesized. Using a codon optimized recombinant nucleoprotein expressed in bacteria, Samudzi* et al.*^[^^24^^] ^detected specific IgG antibody against 12 CCHF patients in South Africa. Sas *et al.*^[^^25^^]^ utilized double antigen ELISA to detect specific antibodies against CCHF virus with multispecies approach and tested three different genotypes of the virus. The CCHF virus has seven district clades with a relatively high diversity at the nucleotide and amino acid levels. It is probable that specific antibodies against one strain would not be able to detect strains from different clades. In this study, five different genotypes circulating in Iran were analyzed, and the IgM capture ELISA could detect all the positive samples in different clades corresponding to 100% specificity.

In our research, the capture ELISA was used to increase the sensitivity of test. Meanwhile, cross-reactivity appears with some viruses, i.e. arboviruses like Dengue, Chikungunya, Zika, and West-Nile. CCHF virus has limited cross-reactivity with other nonpathogen viruses, like Hazara, in human. The non-pathogenonic character of this virus in human decreases the chance of cross-reactivity. In conclusion, we believe that our recombinant nucleoprotein can be used in any ELISA-based technique to detect human IgM antibody in any lab in endemic regions. 
